# Influence of Substance Use Disorder on Treatment Retention of Adult-Attention-Deficit/Hyperactive Disorder Patients. A 5-Year Follow-Up Study

**DOI:** 10.3390/jcm10091984

**Published:** 2021-05-05

**Authors:** Alessandro Pallucchini, Marco Carli, Angelo G. I. Maremmani, Marco Scarselli, Giulio Perugi, Icro Maremmani

**Affiliations:** 1 PISA—School of Clinical and Experimental Psychiatry, 56100 Pisa, Italy; pallucchini.a@gmail.com (A.P.); angelo.maremmani@uslnordovest.toscana.it (A.G.I.M.); 2 Department of Clinical and Experimental Medicine, School of Clinical Pharmacology, University of Pisa, 56100 Pisa, Italy; carlimarco@outlook.it; 3 Association for the Application of Neuroscientific Knowledge to Social Aims (AU-CNS), 55045 Pietrasanta, Italy; 4 North-Western Tuscany Local Health Unit, Department of Psychiatry, Tuscany NHS, Versilia Zone, 55049 Viareggio, Italy; 5 Department of Translational Research and New Technologies in Medicine and Surgery, University of Pisa, 56100 Pisa, Italy; marco.scarselli@med.unipi.it; 6 Department of Clinical and Experimental Medicine, Section of Psychiatry, University of Pisa, 56100 Pisa, Italy; giulio.perugi@med.unipi.it; 7 Vincent P. Dole Dual Diagnosis Unit, Department of Neurosciences, Santa Chiara University Hospital, University of Pisa, 56100 Pisa, Italy; 8 G. De Lisio Institute of Behavioral Sciences, 56100 Pisa, Italy; 9 Saint Camillus International University of Health and Medical Sciences—UniCamillus, 00131 Rome, Italy

**Keywords:** adult attention-deficit/hyperactivity disorder, substance use disorder, survival in treatment

## Abstract

Attention-Deficit/Hyperactivity Disorder (ADHD) is the most widespread neurodevelopmental disorder, and it still persists into adulthood in 2–6% of the population. Psychiatric comorbidities are very common in adult ADHD (A-ADHD) patients; in particular, Substance Use Disorder (SUD) is found in 40% of these patients. Co-occurrence of ADHD and SUD is described as detrimental to clinical outcome by many authors, while only a few studies describe good clinical results in A-ADHD-SUD patients when they were treated for ADHD, both for the efficacy and the compliance of patients. In this study we tested to determine whether SUD can influence the treatment outcome of A-ADHD patients by correlating lifetime, past and current substance use in A-ADHD patients with their outcome (retention rate) during a 5-year follow-up of patients treated with stimulant and non-stimulant medications, using Kaplan–Meier survival analysis with overall and pairwise comparison. The association between demographic, symptomatological and clinical aspects with retention in treatment, adjusting for potential confounding factors, was summarized using Cox regression. After 5 years of observation, the cumulative treatment retention was 49.0%, 64.3% and 41.8% for A-ADHD patients without lifetime SUD (NSUD/A-ADHD), A-ADHD with past SUD (PSUD/A-ADHD) and A-ADHD with current SUD (CSUD/A-ADHD), respectively. Overall comparisons were not significant (Wilcoxon Rank-Sum (statistical) Test = 1.48; df = 2; *p* = 0.477). The lack of differences was confirmed by a Cox regression demonstrating that the ADHD diagnosis according to DIVA, gender, education, civil status, presence of psychiatric comorbidity, and psychiatric and ADHD familiarity; severity of symptomatological scales as evaluated by WHODAS, BPRS, BARRAT, DERS, HSRS, and ASRS did not influence treatment drop-out (χ2 22.30; df = 20 *p* = 0.324). Our A-ADHD-SUD patients have the same treatment retention rate as A-ADHD patients without SUD, so it seems that substance use comorbidity does not influence this clinical parameter.

## 1. Introduction

Attention-Deficit/Hyperactivity Disorder (ADHD) is the most common neurodevelopmental disease, and about 2–6% of the adult population fulfill the criteria for the disorder [1]. Hyperactivity symptoms usually go into remission, whereas inattention and impulsivity persist into adulthood [2]. Co-occurring disorders are frequent in adult-ADHD (A-ADHD) patients; in particular, about 40% of A-ADHD patients also have Substance Use Disorder (SUD) [3,4,5]. The relationship is bidirectional, with 25–30% of adults suffering from SUD who also have ADHD [6]. ADHD shows some features, such as impulsivity, sensation-seeking behavior, and difficulty in modulating reward and gratification [7], all of which give rise to a propensity towards substance use and a major risk for illicit or licit involvement with drug-taking [8]. The impairment of executive functioning and poor delayed reward may lead subjects with ADHD as well as those with SUD, to have a higher likelihood of trying substances than their peers without ADHD [9,10]. From a neurobiological perspective, delayed gratification is mediated by tonic dopaminergic signaling in striatal and prefrontal regions, while immediate processes are predominantly driven by phasic dopamine firing [9]. These neural circuits are deficient in ADHD due to the impairment of dopaminergic physiology [11] and drugs of abuse, such as cocaine, amphetamine, and methamphetamine increase the dopaminergic transmission, especially in the nucleus accumbens [12]. In line with this, stimulant drugs are used by a subgroup of patients as a self-treatment, in accordance with the self-medication hypothesis [13], and possibly reduce the restlessness, the inattention and the emotional lability [9,14,15]. In a recent study, we demonstrated that A-ADHD cocaine users strongly reduced their addiction behaviors once treated with methylphenidate (MPH), and the reduction was proportional to the improvement in A-ADHD symptomatology [16]. Similarly, the use of alcohol, cannabinoids, and heroin by ADHD adolescents was reported to relieve the specific psychopathological features derived from the disorder [17,18].

On the other hand, ADHD in individuals with SUD complicates the diagnosis and treatment [19]. Ideally, ADHD medication for co-occurring ADHD and SUD should appropriately alleviate ADHD impulsivity and restlessness, while improving cognitive control over substance abuse [20]. Instead, many authors reported that the good results achieved with stimulants in ADHD are not matched in the presence of SUD comorbidity, and this could be partly explained by lower treatment retention in ADHD-SUD patients. Comorbidity with SUD was shown to be a predictor of a lower level of treatment retention in a retrospective study [20]. Most randomized clinical trials on the treatment of ADHD-SUD patients show excessive variability in retention rates, with confusing and unclear results [20]. Long-term pharmacotherapy is fundamental in ADHD patients and the dropout may be associated to suboptimal symptoms control, so treatment retention is considered a positive clinical indicator [21]. In contrast with a majority of studies, the prescription of stimulants was shown to improve ADHD symptoms and retention in treatment in some subgroups of SUD patients with ADHD [22]. A beneficial effect of administering ADHD medications in reducing the frequency of dropout by patients in treatment has recently been reported [23] and the use of stimulants, despite their possible addictive potential, should be considered for these patients, considering the good clinical efficacy reported [16]. This evidence of a better outcome in SUD patients could be related to a better understanding of SUD therapy for specific medical centers, which not only rely on detoxification but also on long term “anti-craving” therapies [16].

As the whole question is still controversial, the aim of the present study is to better understand if the co-occurrence of SUD in a population of A-ADHD patients can influence treatment retention and/or rate of dropout from medication treatment in an outpatient psychiatric setting that has extensive experience in the treatment of Dual Disorder patients.

## 2. Materials and Methods

### 2.1. Therapeutic Setting

Most of our SUD/A-ADHD patients were recruited at the Second Psychiatric Unit of Santa Chiara University Hospital, Pisa, which includes a specialized Dual Disorder Unit where some patients have only SUDs.

The setting of the SUD treatment featured the following characteristics: outpatient treatment; easy access to therapy; delivering various different types of intervention for addictive disorders and related problems (opioid, GABAergic, antiglutammatergic, and pro-dopaminergic medications for long-term treatment, general medical care, counselling, rehabilitative services, and psychological–psychiatric care); participation of patients in the determination of medication dose and knowledge of the dose dispensed; urine specimens collected on a weekly basis, and analyzed for morphine and cocaine (availability of 1–3 results per month).

### 2.2. Design of the Study

Research information on patients included in the present study came from the A-ADHD Clinic (A-ADHD outpatient clinic) study, a cohort study including information on patients admitted to outpatient treatment for A-ADHD at the Second Psychiatric Unit of Santa Chiara University Hospital, University of Pisa, and at the Department of Neurology and Psychiatry of the Sapienza University of Rome, Italy. For the purposes of the present study, data collected at the time of enrolment into the study (baseline) and at the last available evaluation, including the status of retention in treatment, were extracted from the A-ADHD-Clinic dataset (Table 1). Once extracted, the information was analyzed following a cross-sectional design to estimate the correlations between substance use and retention in the treatment of A-ADHD patients.

All the subjects examined signed an informed consensus document that allowed them to participate in the A-ADHD-Clinic study. Both the consent form and the experimental procedures were approved by the ethics committee of the University of Pisa (study ID: 14003; code: ADHD-MOOD), in accordance with internationally accepted criteria for ethical research. Aims, data gathered, and the future perspectives of this study are summarized in Table 1.

### 2.3. Sample

The sample consisted of 118 A-ADHD outpatients according to the DSM-5 criteria; 84 (71.2%) patients were males and 34 (28.8%) females. At the time of recruitment, the average age of the sample was 27.19 ± 10.2 years (minimum 17, maximum 54). The duration of the education received was 13.55 ± 3.2 years; 106 (89.8%) patients were single.

Sixty-seven (56.8%) patients have been treated with methylphenidate (MPH) (mean dosage 30 mg/die, 10–60 ranging), while 51 (43.2) have taken non-stimulant medications (Atomoxetine (ATM) or Bupropion). Atomoxetine was used at mean dose of 40 mg/die (min 25 mg/die; max 80 mg/die). Bupropion was used in 5 (4.2%) subjects at dosages of 150 mg/die. At the baseline, treatment was decided based on the clinical status of the patients. Tendentially to the more impulsive ones and with SUD MPH was preferred having a greater effect on the ventral striatum (more effective on craving). Ninety-five (80.5%) patients showed a psychiatric comorbidity, 86 (72.9%) first degree psychiatric familiarity, 19 (16.1%) first degree SUD familiarity, and 45 (38.1%) first degree ADHD familiarity.

Forty-nine (41.5%) had never had a comorbid SUD (NSUD/A-ADHD); 14 (11.9%) had only had that condition in the past (PSUD/A-ADHD), and 55 (46.6%) had it at treatment entry (CSUD/A-ADHD). So current/past comorbid SUD was reported for 69 (58.5%) A-ADHD patients.

### 2.4. Instruments

The diagnosis of ADHD was made according to DSM-5 criteria and confirmed using the Italian version of the semi-structured, clinician-administered Diagnostic Interview for ADHD in adults (DIVA 2.0) [24]. Whenever possible, a caregiver/family member of the patient participated in the interview to provide retrospective and collateral information. The interview is made up of three parts: 9 criteria for attention deficit, 9 criteria for hyperactivity-impulsivity and a last part evaluating the age of onset and the level of impairment. The presence/absence of each item was investigated both in childhood/adolescence and adulthood. If collateral information provided by the caregiver was not available, the diagnosis was based on the patient’s recollection only.

The clinical evaluation was based on the following scales:

Adult ADHD Self-Report Scale (ASRS) [25]: this 18-question scale was used to assess the frequency of self-reported adult ADHD symptoms derived from the DSM-IV criteria. Answers are expressed on a Likert scale, ranging from 0 (“never”) to 4 (“very often”) and higher total score indicates significant levels of ADHD symptoms.

Barratt Impulsiveness Scale (BIS-11) [26]: this is a self-report questionnaire designed to investigate the personality/behavioral construct of impulsiveness, and is considered the gold standard measure of impulse control. The BIS-11 consists of 30 questions and answers are expressed as a range from 1 (“never/rarely”) to 4 (“almost always/always”). The scale is structured following a factorial analysis in six first-order factors (attention, cognitive instability, motor, perseverance, self-control, and cognitive complexity) and three second-order ones (attentional, motor, non-planning impulsiveness).

Brief Psychiatric Rating Scale (BPRS) [27,28]: this 18-item tool is commonly used by the clinician to assess current psychopathology and symptom severity.

Difficulties in Emotion Regulation Scale (DERS) [29]: this is a 36-item self-evaluation scale of six subdomains of emotion regulation (awareness, clarity, goals, impulse, non-acceptance, strategies). Answers range from 1 (“almost never”) to 5 (“almost always”) and higher total DERS scores reflect greater difficulty in regulating emotions.

Hypomania Check List-32 (HCL-32) [30].: this scale is used to evaluate lifetime hypomanic symptoms; it consists of 32 questions with yes/no answers and has a two-factor structure (active/elated hypomania and risk-taking/irritable hypomania).

WHO Disability Assessment Schedule (WHODAS 2.0) [31]: this 36-item questionnaire is used to explore self-reported functioning and disability in six major life domains: cognition, mobility, self-care, getting along, life activities, and participation. Answers are given on a Likert scale ranging from 1 (“no difficulty”) to 5 (“extremely difficult/cannot do”).

### 2.5. Data Analysis

Patients were divided into two groups for the first analysis, based on SUD co-occurrence and no SUD co-occurrence. Comparison of retention in treatment was performed using the Kaplan–Meier survival analysis and the Wilcoxon Rank-Sum (statistical) Test for comparison between the survival curves. For the purpose of this analysis, the term ‘censored observations’ (positive outcome) refers to patients who were still in treatment at the observation point, or were leaving treatment for reasons unrelated to the treatment itself (e.g., when patients were moving to other towns/cities). We considered it to be a negative outcome (terminal event) when a patient dropped out of the treatment not presenting to the scheduled follow-up. For positive outcome the following characteristics have been added: “if symptoms are present, they are transient and expectable reactions to psychosocial stressors (e.g., difficulty in concentrating after family arguments): no more than slight impairment in social, occupational, or school functioning (e.g., temporarily falling behind in schoolwork). In the “negative” outcome these criteria were not met. We compared survival rates for study participants according to the presence of a comorbid SUD. The association between SUD comorbidity and retention in treatment was summarized using Cox regression. In our model we included sociodemographic, clinical, and symptomatological variables that may act as confounding factors.

## 3. Results

During the first year of follow-up 39 out of 118 patients dropped out of the treatment, so that cumulative survival was 0.67. After a further 20 subjects had dropped out during the second year, cumulative retention was 0.46. Three patients left the program during the third year of follow-up, leaving cumulative survival at 0.41. Notably, none of the patients who reached the third year of follow-up dropped out at any time in the fourth or fifth year.

Patients still in treatment at the end of the 5-year follow-up were 24 out of 49 (49.0%), 9 out of 14 (64.3%), and 23 out of 55 (41.8%) for NSUD/A-ADHD, PSUD/A-ADHD, and CSUD/A-ADHD, respectively (Table 2). No statistically significant difference in the percentage of patients still in treatment was reported by using the Wilcoxon Rank-Sum (statistical) Test. Considering observation time related to the first, second, and third year of treatment does not modify these results.

**Figure 1:**
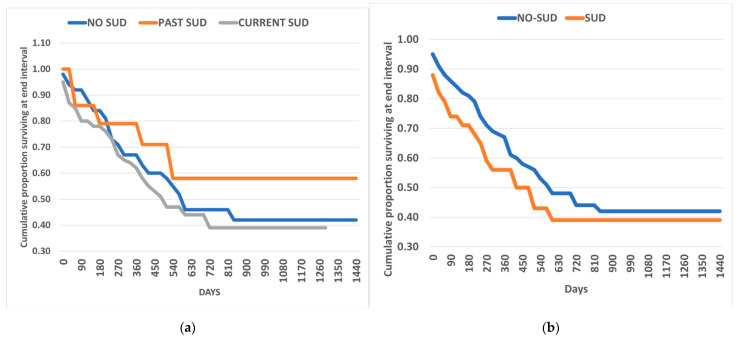
Retention in treatment of A-ADHD patients treated with A-ADHD medications according to Past SUD, Current SUD, and no-SUD (**a**) and with and without lifetime SUD comorbidity (**b**).

Kaplan–Meier survival analysis again showed the lack of statistically significant differences in dropout rates during the 5 years of observation. Trends over time for patients with ADHD with and without SUD are shown in Figure 1a. In Figure 1b, the analysis is further extended for the ADHD-SUD group, which is divided into past SUD and current SUD.

Following the addition of demographic, symptomatological and clinical aspects as potential confounding factors in a Cox model of proportional hazards, once again no association was found between the presence of a SUD and the retention in treatment of our patients (Table 3) (χ^2^ 22.30; df = 20 *p* = 0.324).

## 4. Discussion

The results obtained in the present study show that our A-ADHD patients with co-occurring SUD do not, in terms of retention in treatment, display a worse clinical outcome than subjects with ADHD but without SUD. No difference in retention was reported either for patients with past-SUD vs. current-SUD vs. non-SUD. These results are controversial when compared with much of the literature on the matter, reporting SUD as a predictive factor for shorter treatment retention and higher dropout rates, together with other negative clinical aspects such as suicidality and hospitalization [32,33,34]. Some studies, however, show that medications for ADHD improve the clinical course of both ADHD and SUD [16,22,23] and may even increase patients’ compliance [22] with treatment, as shown by our data. Both the clinical indications and the neurobiology of ADHD and SUD suggest that, in fact, medications for these disorders should have beneficial effects on the behavior of patients and, possibly, on their compliance with treatment [9]. In addition, in our clinic, there is a specialized team for the treatment of SUD patients. The strength of our program for SUD patients is made evident by the long-term treatment of addiction, without recourse to any immediate detoxification of the patients. Our guiding principles in applying the treatment are in line with the use of agonist medications for the substance being used, whenever feasible. In other cases, we administered anti-craving medications on a long-term basis. It should be noted that in the case of cocaine use, stimulant drugs sometimes act as anti-craving drugs.

The chances of a positive effect of medications on both ADHD and SUD, as well as a higher frequency of retention in treatment, are higher with an increasing severity of ADHD features [35,36]. In line with these findings, the positive retention rate we describe could be related to the high level of ADHD symptomatology, as reported by the median value (23) of the ADHD index in our sample. The severity of ADHD in our sample also influenced the rate of treatment engagement, with many patients who missed the first medical assessment or refused therapies immediately.

Neurobiological studies have consistently shown that prolonged exposure to psychoactive drugs leads to neuroadaptive changes in various brain circuits, triggering intrusive thinking (craving), and compulsive behavior in facing dilemmas on the use of substances [10,37,38,39]. The exact influence of these changes on the pathophysiology of ADHD is unknown, but it has been demonstrated that a subgroup of patients uses drugs as self-medication [40]. This is true of stimulants, which act on ADHD core symptoms, so reducing mental and physical restlessness, inattention, and emotional alteration arising from dopaminergic dysregulation [13,41]; but is also true of other substances such as alcohol, cannabinoids, and heroin, which relieve specific psychopathological aspects derived from the disorder [17,18]. The self-therapeutic use of substances could explain the absence of negative effects of SUD on treatment retention in our A-ADHD patients.

What is more, various studies highlight the fact that the chronic use of psychoactive substances reduces the therapeutic efficacy of drugs commonly used for ADHD treatment and the length of the treatment itself [20,42]. One interpretation of these data is that patients with SUD might have developed a tolerance to central stimulants. Our findings are inconsistent with this viewpoint, considering that more than a third of these patients have been using psychoactive substances for a long time. It must, however, be added that higher dosages of ADHD drugs often compensate for the alteration in sensitivity to central stimulants, resulting in a better compliance with long-term treatment in patients who have ADHD and/or SUD [22,43,44]. The integration to medication with cognitive behavioral therapy could have some chance to increase patient’s quality of life and treatment retention, but data on A-ADHD/SUD patients are lacking [45].

Generally speaking, the efficacy of ADHD treatment is negatively influenced by the additional burden of psychiatric comorbidity [35,46,47]. However, the comorbidity of SUD could be an exception and improve the response to such medications [46,47]; our group also recently showed a high efficacy of stimulants in cocaine-addicted patients with ADHD [16]. On the other hand, the use of stimulants was correlated with a much lower dropout rate in ADHD-SUD patients treated for SUD [23]. This bidirectional relationship adds evidence to a possible clinical benefit of medication in both ADHD and SUD when they co-occur, so positively influencing treatment retention in A-ADHD-SUD patients.

First, this study has several limitations. First of all, the sample size was not big enough to generalize our findings. All the patients evaluated were never diagnosed or treated for ADHD before adulthood and presented with a severe clinical picture. To confirm our data a wider sample should be enrolled. The second limitation is the self-assessment of several features, such as impulsivity, emotional dysregulation, and overall functioning, which may lead to reporting biases.

The strength is that our sample included patients treated in a dual disorder unit, very different from locations in which psychiatric units and addiction units work independently, resulting in poor communication between the two. The collaboration should be encouraged and the treatment of both aspects of patient psychopathology should be addressed also in non-dual disorder settings.

## 5. Conclusions

In our Dual Disorder Unit, A-ADHD-SUD patients have the same treatment retention rate as A-ADHD patients without SUD, so substance use comorbidity seems to exert no influence on this clinical parameter, at least when patients are treated in a dual disorder therapeutic setting.

## Figures and Tables

**Table 1:** Aim and data gathered.

• Aim
○ To study the effect of comorbid SUD in A-ADHD outpatients treated in a dual disorder treatment setting
• Data gathered
○ Demographics
▪ Age
▪ Gender
▪ Education
▪ Civil Status
○ Past/Current SUD
▪ THC
▪ Cocaine
▪ MDMA
▪ Amphetamines
▪ Alcohol
▪ Benzodiazepines
▪ Opioids
○ Clinical evaluation
• Length of treatment
• Retention in treatment status
• Still in treatment with transient symptoms and expectable reactions to psychosocial stressors
• Leaving treatment for reasons unrelated to the treatment itself
• Not presenting for the scheduled follow-up
• Treatment with stimulants (MPH)/atomoxetine (ATM)/buproprion
• Psychiatric comorbidity
• First-degree psychiatric familiarity
• First-degree SUD familiarity
• First-degree ADHD familiarity
○ Symptomatology
▪ BARRAT severity (total score)
▪ BPRS severity (total score)
▪ DERS severity (total score)
▪ HSRS severity (total score)
▪ WHODAS severity (total score)

**Table 2:** Retention in treatment of 118 A-ADHD outpatients.

Comparison Group		N	Dropout	In Treatment	% In Treatment	Wilcoxon Statistic	df	*p*
1 vs. 2	NSUD/A-ADHD	49	25	24	49.0	0.351	1	0.552
	PSUD/A-ADHD	14	5	9	64.3			
1 vs. 3	NSUD/A-ADHD	49	25	24	49.0	0.605	1	0.437
	CSUD/A-ADHD	55	32	23	41.8			
2 vs. 3	PSUD/A-ADHD	14	5	9	64.3	1.253	1	0.263
	CSUD/A-ADHD	55	32	23	41.8			

1-NSUD/A-ADHD = Adult-Attention Deficit Hyperactive Disorder without lifetime Substance Use Disorder. 2-PSUD/A-ADHD = A-ADHD = Adult-Attention Deficit Hyperactive Disorder with Past Substance Use Disorder. 3- CSUD/A-ADHD = A-ADHD = Adult-Attention Deficit Hyperactive Disorder with Current Substance Use Disorder.

**Table 3:** Correlation between negative outcome to treatment and associate covariates in 118 A-ADHD study participants.

Variables	B	Exp(B)	Lower	Upper	Sig
Demographic					
Age	0.02	1.02	0.98	1.05	0.377
Gender (Female)	−0.40	0.67	0.30	1.49	0.326
Education					0.381
Primary School		1.00			
Middle School	−0.08	0.92	0.11	7.91	0.940
High School	−0.46	0.63	0.07	5.44	0.675
University	−1.03	0.36	0.04	3.67	0.387
Civil status (not single)	−0.47	0.63	0.20	1.93	0.415
Symptomatology					
ASRS severity	0.01	1.01	0.98	1.03	0.709
BARRAT severity	0.01	1.01	0.98	1.05	0.367
BPRS severity	0.02	1.02	1.00	1.05	0.076
DERS severity	−0.01	0.99	0.97	1.00	0.067
HCL-32 severity	−0.05	0.95	0.89	1.02	0.132
WHODAS severity	0.00	1.00	0.98	1.02	0.922
Clinical aspects					
Psychiatric comorbidity	−0.48	0.62	0.28	1.34	0.223
First-Degree Psychiatric Familiarity	0.09	1.09	0.52	2.27	0.817
First-Degree SUD Familiarity	0.68	1.98	0.93	4.22	0.077
First-Degree ADHD Familiarity	−0.36	0.70	0.35	1.38	0.299
Treatment (stimulants vs. no-stimulants)	−0.12	0.89	0.49	1.61	0.693

Statistics: χ^2^ 22.30; df = 20 *p* = 0.324.

## Data Availability

The data presented in this study are available on request from the corresponding author.
